# Everolimus‐induced gastric antral vascular ectasia in advanced renal cancer

**DOI:** 10.1002/iju5.12221

**Published:** 2020-09-21

**Authors:** Kenichi Hata, Keiji Yasue, Gen Ishii, Takahiro Kimura, Shin Egawa

**Affiliations:** ^1^ Department of Urology Atsugi City Hospital Atsugi Kanagawa Japan; ^2^ Department of Urology Jikei University School of Medicine Minato City Tokyo Japan

**Keywords:** adverse event, everolimus, gastric antral vascular ectasia, GAVE, renal cancer

## Abstract

**Introduction:**

Although several medical, endoscopic, and surgical treatment options are available, the management of gastric antral vascular ectasia remains clinically challenging. We report a case of gastric antral vascular ectasia due to everolimus use in a patient with advanced renal cancer.

**Case presentation:**

A 71‐year‐old man was diagnosed with right‐sided renal cancer and multiple lung metastases. In the period of everolimus as third‐line therapy, endoscopy of the upper gastrointestinal tract revealed everolimus‐induced gastric antral vascular ectasia. Endoscopic argon plasma coagulation and variceal ligation were repeated seven times within a month of everolimus cessation. Subsequently, an antrectomy was performed; his postoperative course was uneventful.

**Conclusion:**

Based on our experience, we believe that an antrectomy is important in the management of mammalian target of rapamycin inhibitor‐related gastric antral vascular ectasia.

**Abbreviations & Acronyms**
APC: argon plasma coagulation
CKD: chronic kidney disease
GAVE: gastric antral vascular ectasia
GIST: gastrointestinal stromal tumor
mTOR: mammalian target of rapamycin
NA: not applicable

**Keynote message**

We observed a case of severe GAVE in a patient receiving everolimus as a third‐line therapy for advanced renal cancer. A surgical treatment approach proved to be vital in the treatment of the mTOR inhibitor‐related GAVE.

## Introduction

GAVE, a relatively uncommon disease, accounts for 4% of non‐variceal upper gastrointestinal hemorrhages, and its clinical presentations differ widely from asymptomatic occult blood loss to overt gastrointestinal bleeding. It is also associated with autoimmune diseases, liver cirrhosis, chronic renal failure, and cardiovascular disease.^1^ Although its pathogenesis remains unclear, it is known to be characterized by typical endoscopic findings including longitudinal rugal folds traversing the gastric antrum and converging on the pylorus, giving the appearance of a “watermelon stomach.” The diagnosis is usually established by this specific endoscopic appearance. Several medical, endoscopic, and surgical treatment options are available for the management of GAVE. However, treatment remains a clinically challenging issue.^2^ GAVE has been reported very rarely in patients receiving several targeted therapies, such as tyrosine kinase inhibitors and mTOR inhibitors.^3^, ^4^, ^5^, ^6^, ^7^, ^8^ To our knowledge, this is the first report of everolimus‐induced GAVE in a patient with advanced renal cancer, which occurred with metastatic renal cancer treatment using everolimus.

## Case presentation

A 71‐year‐old man, with a known history of hypertension and CKD, was diagnosed with right‐sided renal cancer and multiple lung metastases. The diagnosis was based on the laboratory findings of hypercalcemia and chronic stage II renal disease.^9^ A histological investigation following a cytoreductive nephrectomy confirmed the presence of a clear cell renal cell carcinoma (T3aN0M1).^10^ Sunitinib (Sutent; Pfizer, New York, NY, USA) was administered orally at a dosage of 50 mg once daily as an initial salvage therapy in 3‐week cycles comprising 2 weeks of treatment followed by 1 week of no treatment. After three cycles, new lesions were detected in his liver and para‐aortic lymph nodes. Sunitinib was substituted with axitinib (Inlyta; Pfizer) (10 mg/day), as second‐line therapy. There was no abnormal esophagogastroduodenoscopy finding for CKD screening on axitinib introduction. Six months later, new metastases appeared in the thoracolumbar spine. The patient underwent external beam radiotherapy to relieve the pain and a combination of denosumab (Ranmark; Daiichisankyo, Tokyo, Japan) and opioids was prescribed.

**Fig. 1:**
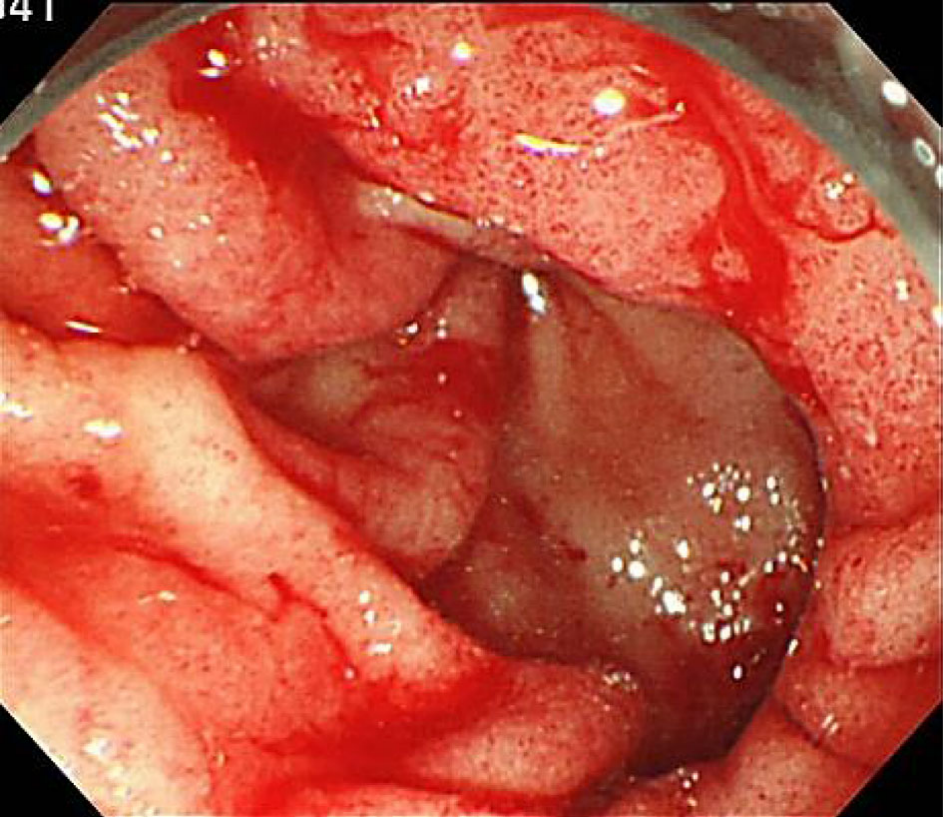
Upper gastrointestinal endoscopy showing lesions typical of a “watermelon stomach.”

Subsequently, everolimus (Afinitor; Novartis, Basel, Switzerland) 10 mg once daily was started. Three months later, he presented to the emergency room of our institution with worsening fatigue, appetite loss, and melena. Blood tests revealed that his hemoglobin level had decreased to 4.5 g/dL (anemia gravis). Treatment with a proton pump inhibitor was started. Upper gastrointestinal tract endoscopy revealed bleeding from scattered mucosal erosions in the gastric antrum, creating a “watermelon stomach” appearance (Fig. 1). The extensive bleeding was controlled using APC and the application of a dry thrombin preparation. Everolimus was discontinued because GAVE was thought to be an adverse drug reaction.

**Fig. 2:**
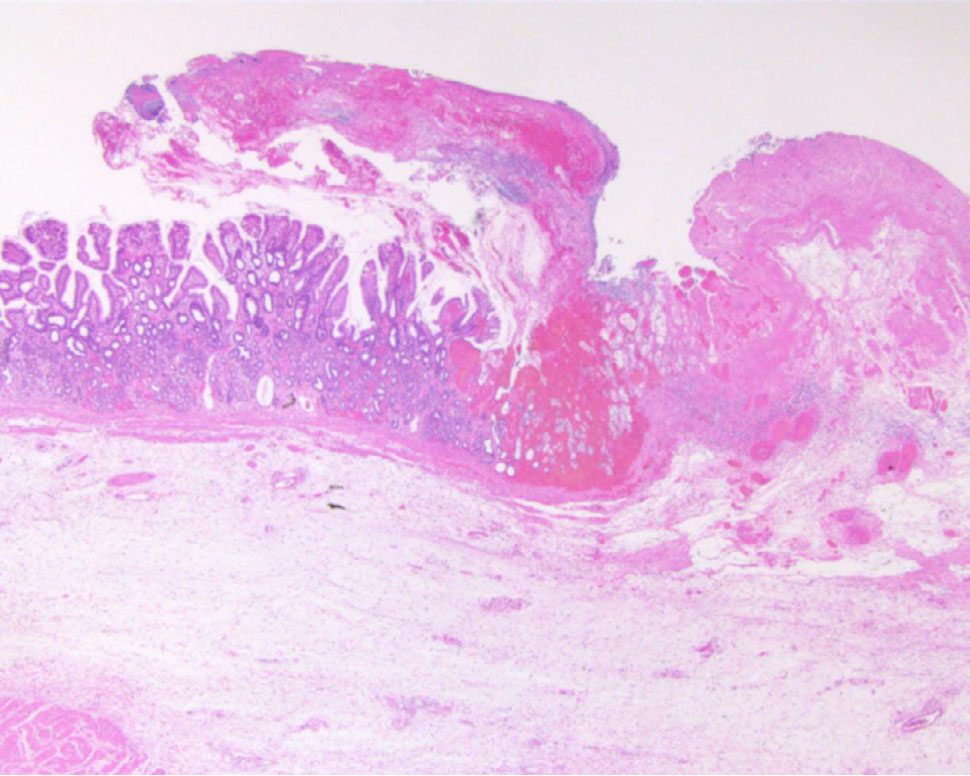
Histopathological examination of the antrectomy specimen showing ectasia of mucosal capillaries, intravascular fibrin thrombosis, and active erosion of the gastric circumference.

Endoscopic APC and variceal ligation were repeated seven times within a month of everolimus discontinuation, along with frequent blood transfusions. Surgical management was decided upon to treat the gastric bleeding and an antrectomy was performed. Histopathological examination of the resected tissue revealed ectasia of mucosal capillaries, intravascular fibrin thrombosis, and active erosion of the gastric circumference (Fig. 2). The postoperative course was uneventful.

## Discussion

Everolimus, an mTOR inhibitor that can be used in the treatment of unresectable and/or metastatic renal cancers, is now approved for clinical use. To the best of our knowledge, only five patients with targeted therapy‐related GAVE on non‐hematological malignancies have been reported (Table 1).^5^, ^6^, ^7^, ^8^ A case of GAVE, which occurred during everolimus treatment for advanced breast cancer, was previously reported by Assi *et al*. This was the first report of everolimus‐related GAVE.^7^ The patient underwent 13 endoscopic procedures before hemostasis was achieved and received 52 units of blood after everolimus was discontinued; the symptoms had significantly improved 2 months later. Fujihara *et al*. reported a case in which life‐threatening GAVE occurred during temsirolimus treatment for advanced renal cancer. The patient was admitted to the department of gastroenterology with worsening fatigue, pallor, and hematemesis 8 weeks after the initiation of targeted therapy. Overall, the patient required APC four times and 38 units of blood within 4 weeks of temsirolimus discontinuation.^6^

**Table 1:** Clinical features of targeted therapy‐related GAVE on non‐hematological malignancies and the response to treatment

No.	Author	Age	Sex	Malignancy	Targeted therapy	Day of onset (days)	Initial symptom	Method of diagnosis	Amount of blood transfusion (units)	Endoscopic procedure	Surgical procedure	Duration of bleeding (days)	Resolution of GAVE	Outcome
1	Saad *et al*.^5^	74	Female	GIST	Imatinib	233	Fatigue Pallor Dyspnea Diaphoresis	Endoscope	4	NA	None	38	Yes	Alive
2	Fujihara *et al*.^6^	40	Female	Renal cell carcinoma	Temsirolimus	56	Fatigue Pallor Hematemesis	Endoscope Histopathological	38	APC	None	60	Yes	Alive
3	Assi *et al*.^7^	48	Female	Breast carcinoma	Everolimus	30	Fatigue Pallor Melena Hematochezia	Endoscope	52	Cyanoacrylate spray APC	None	34	Yes	Alive
4	Abu‐Amna *et al*.^8^	68	Female	GIST	Imatinib	60	Anemia	Endoscope	Not mentioned	APC	None	1000	Yes	Alive
5	Present case	71	Male	Renal cell carcinoma	Everolimus	90	Fatigue Appetite loss Melena	Endoscope Histopathological	30	APC Variceal ligation Dry thrombin	Antrectomy	32	Yes	Alive

No previous study has clearly established the relationship between targeted therapy and the pathogenesis of targeted therapy‐related stomatitis and GAVE. However, the pathogenesis of GAVE is suspected to be associated with the mechanical stress caused by altered gastric motility and gastric dysfunction, which induces chronic mucosal trauma and subsequent submucosal fibromuscular hyperplasia and dilation of mucosal capillaries.^1^ Furthermore, everolimus is known to increase gastrointestinal motility, leading to common side effects, including nausea, vomiting, and diarrhea.^11^ Increased gastrointestinal motility may contribute to the development of everolimus‐induced GAVE. Moreover, in percutaneous coronary intervention, everolimus, a second‐generation polymer coating, is used as a coronary stent because it can up‐regulate genes related to thrombosis, inflammation, and vasoconstriction.^12^ Everolimus is widely used for the prevention of organ rejection after various transplantations, renal angiomyolipoma, and subependymal giant cell astrocytoma associated with tuberous sclerosis complex and advanced neoplastic diseases, as it functions as an active immunosuppressive, anti‐thrombogenic, and antiproliferative agent.^13^

Over the last two decades, various therapeutic options have been proposed for GAVE, including several medical (e.g. corticosteroid, estrogen‐progesterone, and thalidomide), endoscopic (e.g. APC, endoscopic band ligation, and radiofrequency ablation), and surgical options. However, the best approach has not been established. Fuccio *et al*. indicated that a surgical approach, most commonly an antrectomy, had established clinical efficacy in the management of GAVE‐related bleeding.^14^

We observed a case of severe GAVE in a patient receiving everolimus for advanced renal cancer, as a third‐line therapy, that resulted in a serious adverse drug reaction. Antrectomy was inevitable after several endoscopic procedures were unsuccessful in managing the gastric bleeding. Further research on the pathogenesis of mTOR inhibitor‐related GAVE in advanced renal cancer is required.

## Author contributions

K. Hata: conception and design; K. Hata, K. Yasue, G. Ishii: acquisition of data; K. Hata, K. Yasue, G. Ishii: analysis and interpretation of data; K. Hata, T. Kimura: drafting of the manuscript; K. Hata, T. Kimura, S. Egawa: critical revision of the manuscript for important intellectual content; T. Kimura, S. Egawa: supervision.

## Conflict of interest

The authors declare no conflict of interest.

## Ethical statement

This report contains a single case. Thus, institutional review board approval was not required. Informed consent was obtained from the subject.

## Data Availability

The authors confirm that the data supporting the findings of this study are available within the article.
